# Genome-wide identification of the auxin response factor (ARF) gene family in *Magnolia sieboldii* and functional analysis of *MsARF5*

**DOI:** 10.3389/fpls.2022.958816

**Published:** 2022-10-05

**Authors:** Mei Mei, Wanfeng Ai, Lin Liu, Xin Xu, Xiujun Lu

**Affiliations:** ^1^Department of Forestry, Shenyang Agricultural University, Shenyang, China; ^2^Biotechnology and Analysis Test Center, Liaoning Academy of Forest Science, Shenyang, China

**Keywords:** auxin response factor, *Magnolia sieboldii*, auxin, embryo, seeds

## Abstract

Auxin plays an essential role in flowering, embryonic development, seed dormancy, and germination. Auxin response factors (ARFs) are plant-specific key transcriptional factors in mediating the gene expression network of auxin signaling. Although ARFs in model plants such as *Arabidopsis* had been well characterized, their identities and potential roles in non-model plants are less studied. Here, we performed genome-wide identification of ARFs in *Magnolia sieboldii* K. Koch, a primitive species with high taxonomic importance and medicinal values. We found 25 ARF genes in *M. sieboldii*, which were widely distributed across multiple chromosomes. Based on sequence similarity, the encoded proteins could be either transcriptional repressors or activators. Gene expression analysis showed a dynamic pattern for many ARFs including *MsARF5* during seed germination. In addition, overexpressing of *MsARF5* showed that it restores many developmental defects in the *Arabidopsis* mutant. Moreover, two phenotypically distinct transgenic *Arabidopsis* lines were obtained, indicating a link between gene expression levels and developmental phenotypes. Taken together, we provided a systematic investigation of the *ARF* gene family in *M. sieboldii* and revealed an important role of *MsARF5* in mediating auxin signaling.

## 1 Introduction

Auxin controls many critical events of plant life including lateral root formation, embryonic development, seed dormancy, and germination. In the model plant *Arabidopsis*, early embryonic patterning is largely dependent on the biosynthesis, transport, and signaling of auxin (Moller and Weijers, 2009). The role of auxin in seed germination has long been recognized due to its antagonistic effect on abscisic acid (ABA) (Rousselin et al., 1992). More recently, a role of indole-3-acetic acid (IAA) in directly regulating seed dormancy and germination has also been discovered (Belin et al., 2009; Wang et al., 2011; Boter et al., 2019).

Genetic studies had identified two families of key transcriptional factors (TFs) in mediating auxin signaling: auxin response factors (ARFs) and Aux/IAA proteins (Woodward and Bartel, 2005; Quint and Gray, 2006). ARFs are a group of plant-specific proteins typically consisting of an N-terminal DNA-binding domain (DBD), a non-conserved middle region (MR), and a carboxyl-terminal dimerization domain (CTD) in mediating protein-protein interaction. ARF proteins regulate the expression of auxin-responsive genes by binding to conserved promoter regions such as the auxin-response element (TGTCTC). They can also form dimers by interacting with Aux/IAA proteins (Tiwari et al., 2003). Interestingly, ARFs could act as either transcriptional activators or repressors depending on the amino acid compositions in the MR region. For instance, auxin contributes to seed dormancy by regulating *ABSCISIC ACID INSENSITIVE 3* (*ABI3*) *via* ARF10 and ARF16, two transcriptional repressors (Liu et al., 2013). In addition, the transcriptional activity of ARFs is also controlled by IAA concentration and different expression levels of ARFs may lead to distinct phenotypes. Thus, the exact role of ARFs in specific physiological contexts deserves detailed investigation.

The first identification of plant ARFs was performed in *Arabidopsis* by yeast one-hybrid (Ulmasov et al., 1997). Following studies identified a total of 23 *ARF* genes in *Arabidopsis* (Okushima et al., 2005). These genes are distributed on all five chromosomes and may encode proteins of high sequence similarity (e.g., *ARF1* and *ARF2*). With the availability of genome sequencing data, genome-wide identifications and functional characterization of *ARFs* had been performed in multiple species. For example, 31 *ARFs* were reported in maize (*Zea mays*) with dynamic expression patterns during embryo development, indicating a potential role of these *ARFs* in regulating pattern formation of the embryo (Xing et al., 2011). Importantly, 18 out of the 31 *ARFs* in maize were putative targets of small RNAs, which can regulate the expression of ARFs by degradation of the transcripts.

Among the 23 *ARFs* in *Arabidopsis*, ARF5/MONOPTEROS (MP) is essential in auxin signaling during embryo patterning (Hardtke and Berleth, 1998; Odat et al., 2014). ARF5/MP mediates the auxin-induced embryonic root initiation and promotes the transport of auxin from embryonic cells to extraembryonic suspensor cells (e.g., precursor cells of the quiescent center) during embryonic cell specification (Rademacher et al., 2012). In addition, *AtARF5* also regulates plant growth and development such as the formation of cotyledons (Cole et al., 2009), apical meristem (Zhao et al., 2010), and vascular tissues in leaves (Donner et al., 2009). Mutation of *ARF5/MP* in *Arabidopsis* results in impaired body axis patterning during the early embryogenic stage (e.g., lack of embryonic root) and decreased complexity in vascular patterning. It has also been demonstrated that mutation of *ARF5/MP* leads to an abnormal expression of *AMP1* (altered meristem program 1) and thus abnormal embryonic differentiation (Vidaurre et al., 2007). Analysis of *ARF5/MP* mutants featuring deletion of different parts of the protein also revealed distinct functions of each domain. For instance, deletion mutations toward the CTD tend to result in a stronger effect phenotypically, suggesting an important role of CTD (e.g., for dimerization) in protein functioning (Liscum and Reed, 2002).

*Magnolia sieboldii* K. Koch is a small deciduous tree of the *Magnolia* family (*Magnoliaceae*). As a relatively primitive angiosperm species with high taxonomic importance, it also has ornamental, aromatic, and medicinal values. Seed germination in *M. sieboldii* is difficult under both natural conditions and even after stratification (Li et al., 2006; Lu et al., 2014; Mei et al., 2017). Recently, a sequencing analysis revealed 13,877 genes showing differential expression patterns between germinated and non-germinated *M. sieboldii* seeds (Mei et al., 2021). Among them, 38 auxin response factors were up-regulated in the germinated seeds, confirming an important role of auxin in mediating seed germination in *M. sieboldii.* However, the molecular mechanism by which auxin and *ARF* genes regulate seed germination and other aspects of plant phenotypes remain unclear.

Here, we performed genome-wide identification of ARFs in *M. sieboldii* and characterized their chromosomal locations, gene structure, protein sequence similarity, and dynamic expression pattern during seed germination. Furthermore, we overexpressed *MsARF5* in *Arabidopsis* and found that it could rescue multiple defects of the *Arabidopsis ARF5/MP* mutant. The impact of overexpressing *MsARF5* on vegetive growth, flowering, and auxin response was also investigated.

## 2 Materials and methods

### 2.1 Sequence identification

ARF proteins in *Arabidopsis* and rice (Wang et al., 2007) were downloaded from TAIR (http://www.Arabidopsis.org) and Phytozome (https://phytozome.jgi.doe.gov/pz/portal.html), respectively. Sequences from other families were obtained according to previous studies that characterized ARFs in *Medicago truncatula* (Shen et al., 2015), apple (Wang et al., 2020), and *Vitis vinifera* (Wan et al., 2014). The obtained sequences were used to search the *M. sieboldii* protein database from NGDC with the BioProject No. of PRJCA009763. In addition, ARF domains (PF06507, PF02362, and PF02309) were used as queries to search the protein family database (PFAM). Top hits were checked by domain analysis using the NCBI CDD tool (https://www.ncbi.nlm.nih.gov/Structure/cdd/wrpsb.cgi) and Pfam (https://pfam.xfam.org).

### 2.2 Bioinformatics analysis

The chromosomal location of each *MsARF* was obtained from the *M. sieboldii* genome database. Motif analysis was performed using MEME Suite (version 4.12.0) with the following parameters: site distribution of 0 or 1 occurrence per sequence, number of motifs of 10, and motif size of 10 to 200 amino acid residues (http://meme-suite.org/tools/meme). Conserved domains were visualized using TBtools (v1.046, https://github.com/CJ-Chen/TBtools) (Chen et al., 2020). Multiple sequence alignment was performed using ClustalX (Larkin et al., 2007). A phylogenetic tree was constructed using MEGA-X (Kumar et al., 2018) with the neighbor-joining (NJ) algorithm and 1000 bootstrap replicates. Hidden Markov model (HMM 3.0) (Finn et al., 2011) software is used to obtain the starting position information of *MsARFs* gene on the chromosome from the genome annotation information of *M. sieboldii*. Homologous replication were evaluated *via* blastp of *MsARF* genes (Ai et al., 2022).

### 2.3 Plant materials and treatments

*M. sieboldii* seeds were collected from the Botanical Garden of Shengyang. Seeds were air-dried and stored at 4°C. Treatment for seed germination was performed as described (Mei et al., 2017). After soaking in water for 72 h, the seed coat was removed. Surface disinfection was performed in 0.25% KMnO_4_ for 30 min. Seeds were then mixed with wet sand (v/v = 1:3) for stratification with the following regimen: low temperature (0 - 10°C) for 30 days, varying temperature (day of 8 - 10°C, night of -5 - 0°C) for 15 days, and high temperature (15°C) for 30 days. For imbibition experiments, seeds were sampled at various time points before (up to 12 h) and after the initiation of imbibition (every 6 h till 72 h). For experiments involving laminated samples, sampling was performed every 15 days for 90 days.

*Arabidopsis* plants including the wild type *Col-0* and the T-DNA mutant of *ARF5* (salk_001058) were obtained from The *Arabidopsis* Information Resource (TAIR) (https://www.arabidopsis.org/index.jsp). Seeds were surface-sterilized with 75% ethanol (30 s) and 1% NaClO (15 min) and then plated on half-strength MS plates. After stratification at 4°C for 3 days, they were maintained at 20-23°C with a 16/8 h of light/dark cycle. Seedlings with 2-4 true leaves were transferred to the soil for further growth.

### 2.4 Gene expression analysis

Real-time quantitative reverse transcription PCR (qRT-PCR) was used for gene expression analysis. Briefly, RNA was isolated using a RNAprep pure plant kit (Tiangen, China). cDNA was synthesized using a PrimeScript™ II 1st strand cDNA synthesis kit (TAKARA, Japan). qRT-PCR was carried out using FastFire qPCR PreMix(SYBR Green) (Tiangen, China) on an ABI Stepone plus system (CA, USA). Primers used were listed in **Table S1**. The 2^-ΔΔCT^ method was used to calculate relative expression levels. Actin was used for normalization. For all experiments, three biological replicates with two technical replicates were performed.

### 2.5 Quantficaiotn of Auxin

Endogenous auxin was determined using an ELISA kit (Hailian Biological Company). Briefly, seed samples were homogenized with 4 mL of PBS (PH7.4) and then centrifuged at 2000 rpm. The supernatant was added to the wells and incubated with enzyme-linked antibodies at 37°C for 60 min. After washing and signal development, absorbance at 450 nm was quantified. A standard curve was built for concentration calculation.

### 2.6 Transgenic lines

Two vectors were created for reverse genetics studies: full-length *MsARF5* driven by either the *35S* promoter (*35S*::*MsARF5*) or its native promoter (*MsARF5*-*Pro::MsARF5*). For *35S*::*MsARF5*, *MsARF5* was amplified by PCR with the introduction of cutting sites of *SalI* and *SacI*. The PCR product was inserted into pRI101. For *MsARF5-Pro*::*MsARF5*, the promotor region of *MsARF5* was fused to the coding sequence of *MsARF5* and then inserted into pCAMBIA3301 as described previously (Lei, 2007). All constructs were confirmed by double digestion and sequencing. Both vectors were introduced to *Agrobacterium tumefaciens* GV3101 strain by the freeze-and-thaw method (Yu et al., 2003).

Transformation of *Arabidopsis* plants was performed as described (Clough and Bent, 1998). Three transformation experiments were conducted with the following constructs and plants (**Table 1**): OE1 (*35S::MsARF5* in the Col-0 background), OE2 (*35S::MsARF5* in the homozygous plant arf-/-), and OE3 (*MsARF5-Pro::MsARF5* in the Col-0 background). Seeds of the transgenic lines were screened on half MS with 30 mg/L Kan and further validated by PCR.

**Table 1:** Transgenic lines used in this study.

Transgenic plants	Background	Description
*OE1*	Col-0	*35S::MsARF5* in the WT Arabidopsis
*OE2*	*arf-/-*	*35S::MsARF5* in the homozygous *arf-/-* mutant
*OE3*	Col-0	*MsARF5-Pro::MsARF5* in the WT Arabidopsis

### 2.7 Phenotypic analysis

Seeds of the third generation of transgenic plants were used. Parameters on plant growth including plant height, ground diameter, rosette diameter, leaf length, leaf width, and internode were measured. In addition, seed germination, bolting, flowering, and senescence were also quantified. Thirty plants were used for each group, and three biological replicates were performed.

### 2.8 Auxin responses

Seeds of the third generation of transgenic plants were used. Half-strength MS medium was prepared either without hormone (for controls) or in the presence of 1 µM of IAA or IBA. Seeds were surface-sterilized and planted on the plate. Observations were made 5 days later under a microscope (Carl Zeiss AG). Forty seeds were used for each group, and three biological replicates were performed.

### 2.9 Statistical analysis

Statistical analysis was performed using the SPSS20.0. Gene expression analysis was performed using TBtools (v1.046). GraphPad Prism 5 was used for data visualization.

## 3 Results

### 3.1 Genome-wide identification of ARF genes in *M. sieboldii*

Homology search revealed that the genome of *M. sieboldii* encode 25 *ARF* genes, which were named based on similarity to that of *Arabidopsis* (**Table 2**). Amon the 25 genes, 23 were localized to 12 chromosomes including HIC_ASM_0 (n = 2), HIC_ASM_1 (n = 4), HIC_ASM_2 (n = 4), HIC_ASM_3 (n = 2), HIC_ASM_4 (n = 3), HIC_ASM_6 (n = 1), HIC_ASM_7 (n = 1), HIC_ASM_9 (n = 1), HIC_ASM_11 (n = 2), HIC_ASM_16 (n = 1), and HIC_ASM_18 (n = 1, **Figure S1**). The other two genes were localized to unassembled chromosomal fragments. In addition, these gene varied greatly in length, as the open reading frame (ORF) ranged from 507 to 3,336 bp. In line with the variation in gene length, there were different numbers of exons (2-14) in the putative *MsARF* genes (2-3 for 4 genes; 4-9 for 6 genes; 11-13 for 5 genes; and 14 for 10 genes).

**Table 2:** Summary of *MsARF* genes encoding ARF proteins in *M. sieboldii*.

Name	ID	Chromosome	ORF	aa	PI	MW kDa	Domain
*MsARF1*	HIC_ASM_1.2985	HIC_ASM_1	2046	680	6.08	75.27	DBD, ARF, CTD
*MsARF2a*	HIC_ASM_4.275	HIC_ASM_4	2589	861	6.07	95.98	DBD, ARF, CTD
*MsARF2b*	HIC_ASM_2.3784	HIC_ASM_2	2580	858	6.53	95.72	DBD, ARF, CTD
*MsARF3a*	HIC_ASM_4.2876	HIC_ASM_4	1215	403	6.65	45.09	DBD, ARF
*MsARF3b*	HIC_ASM_2.292	HIC_ASM_2	2484	826	5.61	90.2	DBD, ARF, CTD
*MsARF4*	HIC_ASM_7.1202	HIC_ASM_7	2415	803	5.26	89.94	DBD, ARF, CTD
*MsARF5*	HIC_ASM_3.2992	HIC_ASM_3	2961	985	5.33	109.28	DBD, ARF, CTD
*MsARF6a*	HIC_ASM_0.3420	HIC_ASM_0	2661	885	6.14	98.36	DBD, ARF, CTD
*MsARF6b*	HIC_ASM_0.3399.1	HIC_ASM_0	2928	974	6.44	109.61	DBD, ARF, CTD
*MsARF8a*	HIC_ASM_3.1528.1	HIC_ASM_3	600	198	5.96	22.31	DBD
*MsARF8b*	HIC_ASM_1.2695.1	HIC_ASM_1	2526	840	5.8	93.52	DBD, ARF, CTD
*MsARF9*	HIC_ASM_2.211	HIC_ASM_2	2106	700	6.29	78.57	DBD, ARF, CTD
*MsARF10a*	001499F.1	001499F	1836	610	9.12	67.84	DBD, ARF
*MsARF10b*	HIC_ASM_2.1300.1	HIC_ASM_2	2112	702	6.54	77.4	DBD, ARF
*MsARF16*	HIC_ASM_4.1755.1	HIC_ASM_4	2268	754	7.54	83.63	DBD, ARF
*MsARF17a*	HIC_ASM_1.733	HIC_ASM_1	1593	529	8.98	57.78	DBD, ARF
*MsARF17b*	HIC_ASM_18.1473	HIC_ASM_18	2019	671	6.58	73.34	DBD, ARF
*MsARF19*	HIC_ASM_6.2304	HIC_ASM_6	3015	1003	7.39	112.4	DBD, ARF
*MsARF25*	HIC_ASM_1.2220	HIC_ASM_1	507	167	10.58	18.68	DBD, ARF
*MsARF26a*	HIC_ASM_9.50	HIC_ASM_9	3336	1110	6.39	123.06	DBD, ARF, CTD
*MsARF26b*	HIC_ASM_11.47	HIC_ASM_11	3333	1109	6.22	122.91	DBD, ARF, CTD
*MsARF27*	001465F.3	001465F	1224	406	7.72	45.23	DBD, ARF
*MsARF28*	001465F.5	001465F	753	249	7.15	27.54	DBD
*MsARF29*	HIC_ASM_11.53	HIC_ASM_11	603	199	6.15	21.95	DBD
*MsARF30*	HIC_ASM_16.1609	HIC_ASM_16	2271	755	5.95	84.44	DBD, ARF, CTD

### 3.2 Putative *Ms*ARF proteins and bioinformatic analysis

**Figure 1:**
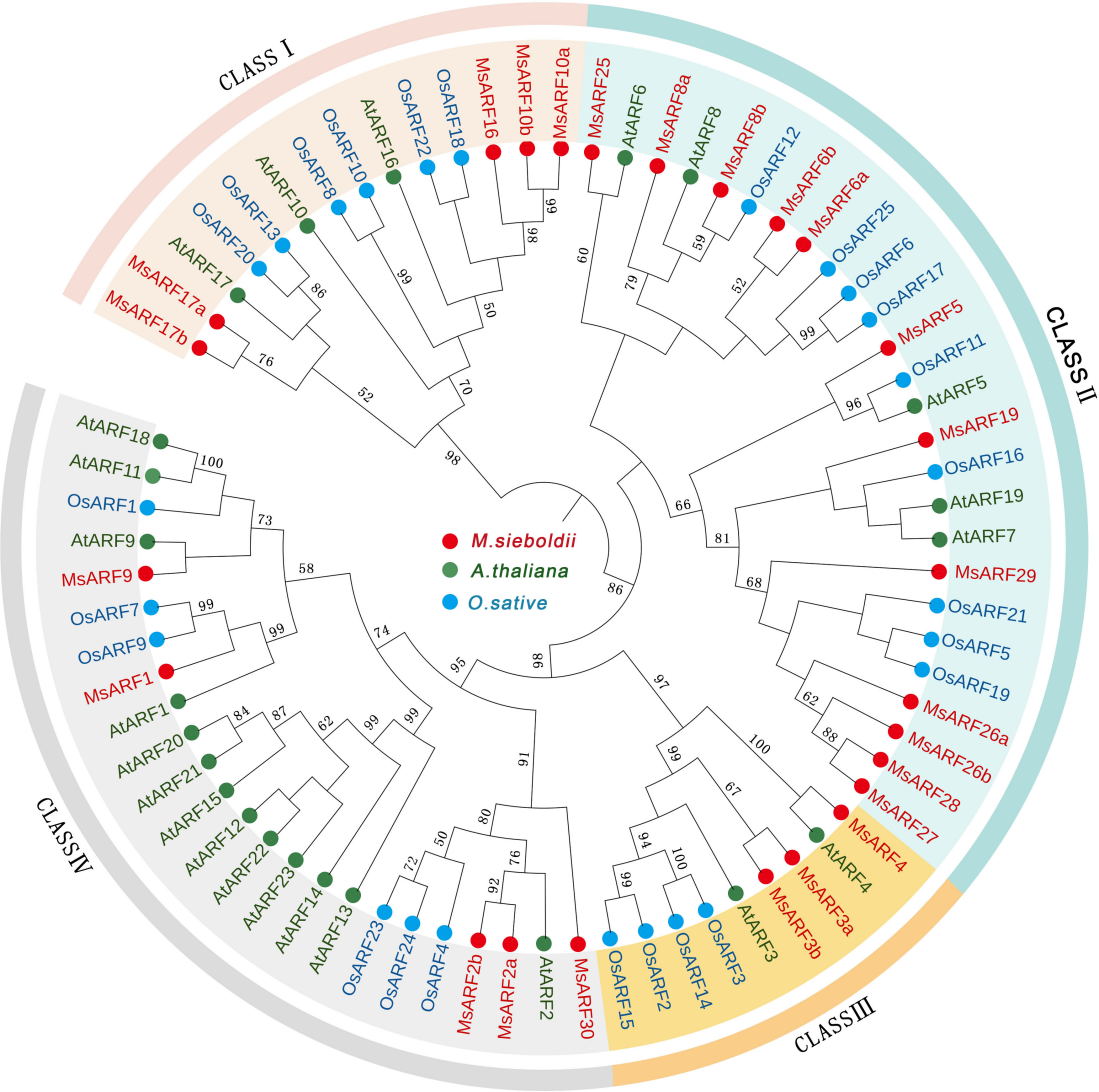
Identification of ARFs in *M. sieboldii* and phylogenetic analysis. The unrooted tree was constructed using MEGA-X with the neighbor-joining method (bootstrap of 1000).

The deduced proteins of these genes ranged from 167 to 1,110 amino acid residues (18.68 to 123.06 kDa) with a wide range of pI values (5.26 to 10.58). Phylogenetic analysis classified 25 *Ms*ARFs into four classes (**Figure 1**). Class I consisted of *Ms*ARF10a/b, *Ms*ARF16, and *Ms*ARF17a/b (homologs of ARF10/16/17 in *Arabidopsis*). Class II consisted of MsARF5, *Ms*ARF6a/b, *Ms*ARF8a/b, *Ms*ARF19, *Ms*ARF25, *Ms*ARF26a/b, and *Ms*ARF27-29 (homologs of ARF5/8 in *Arabidopsis*). Class III consisted of *Ms*ARF3a/b, and *Ms*ARF4 (homologs of ARF3/4 in *Arabidopsis*). Lastly, class IV consisted of *Ms*ARF1, *Ms*ARF2a/b, *Ms*ARF9, and *Ms*ARF30 (homologs of ARF1/2 in *Arabidopsis*).

*Ms*ARFs can also be classified based on the presence of a C-terminal dimerization domain (CTD) and the nature of the middle region (MR), which could function as either transcriptional repression or activation domain (**Table 2**). Thus, while seven *Ms*ARFs (*Ms*ARF5, *Ms*ARF6a/b, *Ms*ARF8b, *Ms*ARF19, and *Ms*ARF26a/b) were identified as putative activator due to the presence of a Q/S/L-rich MR domain, seven *Ms*ARFs (*Ms*ARF1, *Ms*ARF2a/b, *Ms*ARF3b, *Ms*ARF4, *Ms*ARF9, and *Ms*ARF30) were identified as putative repressors since they harbor a P/G-rich MR domain. In addition, three *Ms*ARFs had only the conserved N-terminal DNA binding domain (DBD) but no CTD or MR. Transcriptional activities for these *Ms*ARFs are unclear. Furthermore, there were eight *Ms*ARFs showing no CTD domain that could be either activators (*Ms*ARF3a, *Ms*ARF10a/b, *Ms*ARF16, and *Ms*ARF17a/b) or repressors (*Ms*ARF25 and *Ms*ARF27).

In addition, 10 conserved motifs ranging from 16 to 50 amino acid residues were identified. Among these motifs, motifs 1-5 belong to DBD (B3 domains), motifs 6-8 belong to ARF (Auxin responsive domains), and motifs 9/10 belong to CTD (AUX/IAA domains). Notably, motifs 1-5 were found in all *Ms*ARFs expect *Ms*ARF10a, *Ms*ARF17a, and *Ms*ARF25. While motifs 6-8 resided in the MR region, motifs 9 and 10 were within the CTD domain. Although the arrangement of these motifs was consistent in most *Ms*ARFs, duplication of some motifs was also found including an extra motif 1 in the MR region of *Ms*ARF5 and an extra motif 7 in *Ms*ARF3b. The presence of these duplicated motifs may indicate their key roles in mediating specific functions.

### 3.3 *MsARF5* is significantly upregulated during seed germination

**Figure 2:**
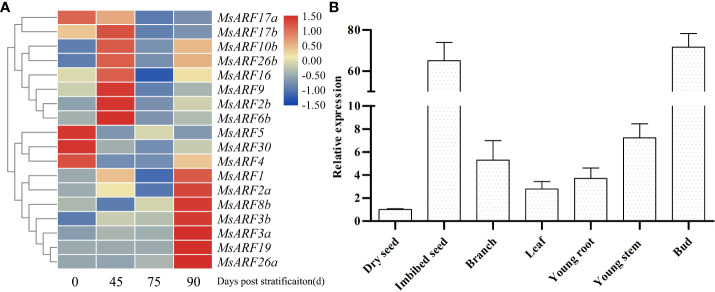
Gene expression analysis of *MsARFs*. **(A)** Expression of 25 *MsARFs* during seed germination. **(B)** Expression of *MsARF5* in various tissues.

Given the important role of ARF during seed development, we examined the gene expression pattern of *MsARF* genes at various seed developmental stages. As shown in **Figure 2A**, multiple genes showed a dynamic expression pattern after various stratification times. The expression pattern of *MsARF10/16*, which are related to root crown development and root tropism, was the same with an up-regulation at 45 d and 90 d. A similar expression pattern was seen for *MsARF17a/b*, indicating a similar structure and function of *MsARF17a/b* compared to that of *MsARF10/16*. It is noteworthy that a negative correlation in expression between *ARF10/16* and *ARF5*, which promote seed dormancy and germination, respectively, was also found. Because of the dominant role of *ARF5* in seed germination (Galstyan and Nemhauser, 2019), we also determined the gene expression pattern of *MsARF5* in various tissues and seed germination stages. We found a relatively low expression in dry seed, a stable expression in shoot and leaf, and a significantly high level in germinating seeds (**Figure 2B**).

**Figure 3:**
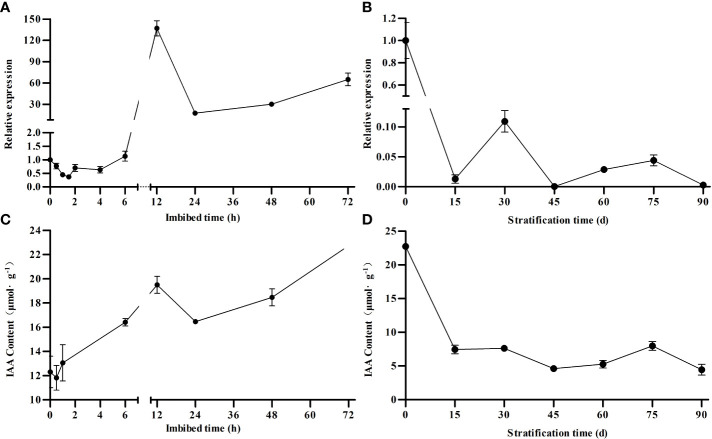
Correlation between *MsARF5* expression and auxin concentration following seed stratification and imbibition. **(A, B)**: relative expression of *MsARF5* following stratification and imbibition. **(C, D)**: auxin concentration following stratification and imbibition.

To further characterize the potential role of *MsARF5* during seed germination, we performed a time-course gene expression analysis after imbibition. The expression of *MsARF5* showed an initial decrease at 1 h after imbibition, followed by a gradual increase, and a sharp increase at 6-12 h after imbibition. Such a high-level expression was maintained up to 72 h after imbibition (**Figure 3A**). Similarly, we profiled the impact of stratification time on *MsARF5* expression and found a general decrease trend (**Figure 3B**). Significant, we also quantified auxin and found a similar trend between the level of auxin and *MsARF5* expression in both events (seed imbibition and stratification in **Figures 3C, D**, respectively). This indicated that *MsARF5* is positively regulated by endogenous auxin in seeds. Given the morphological changes in the embryo during stratification, it was speculated that *MsARF5* is involved in embryonic development after maturation (0-30 days after stratification), cell elongation, and rapid growth of seed embryos (60-75 days).

### 3.4 Overexpressing *MsARF5* in *Arabidopsis* rescues the *Arabidopsis arf5* mutant in vegetative growth

**Figure 4:**
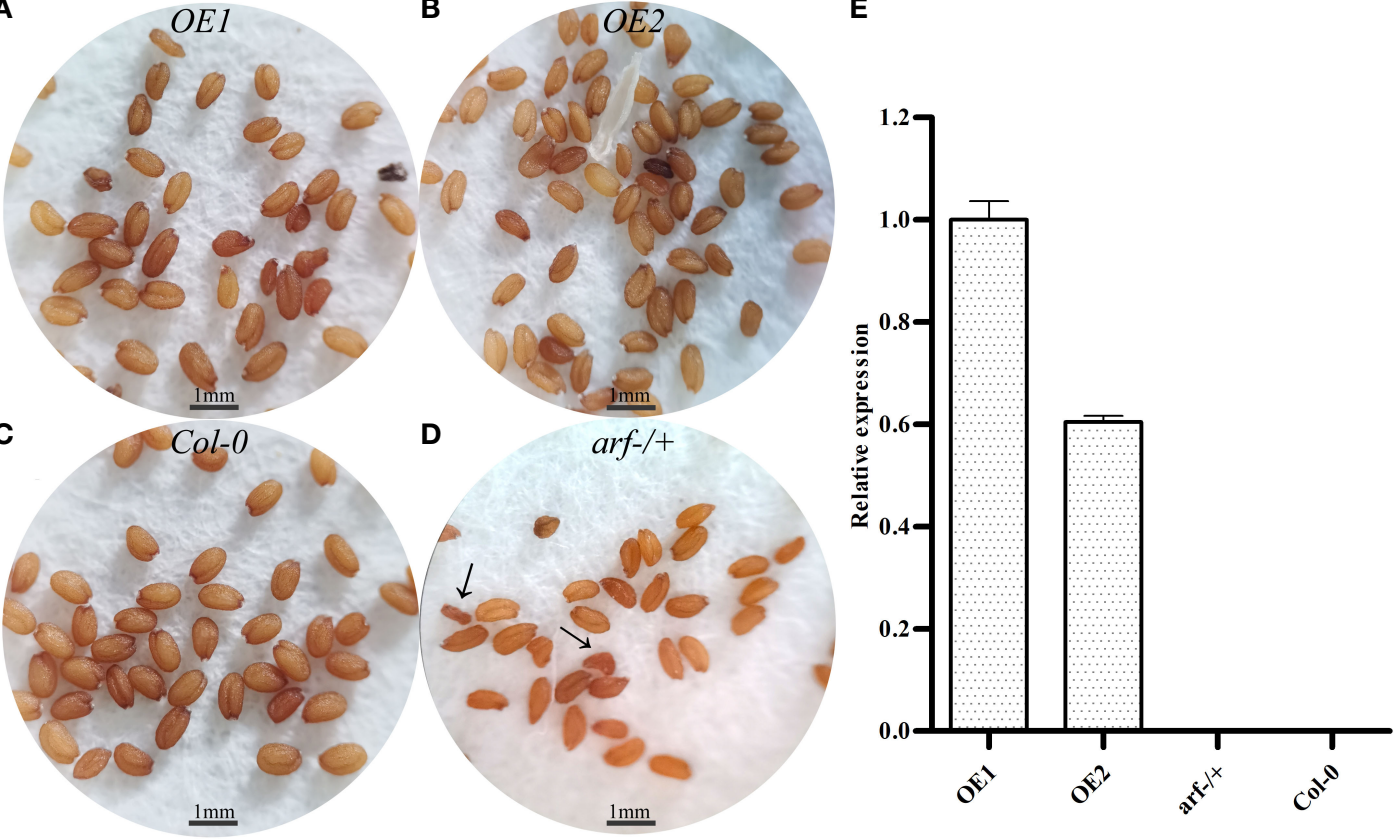
Phenotypes of transgenic *A. thaliana* overexpressing *MsARF5*. **(A-D)**: Seed morphology of OE1, OE2, WT (*Col-0*) and *arf-/+* (heterozygous mutant of *AtARF5*) **(E)**: Results on RT-qPCR of transgenic plants overexpressing.

For further functional investigation, we created transgenic plants overexpressing (OE) *MsARF5* in the *Col-0 A. thaliana*. And the *MsARF5* expression results of the transgenic lines were obtained (**Figure 4E**).The expression of *OE1* was significantly higher than that of *OE2*, and *MsARF5* was not detected in *Col-0*, *arf-/-* or *arf+/-*. It is speculated that the expression of *MsARF5* may be affected by *AtARF5*. The phenotypic difference of *OE1/OE2* may be related to the expression of *MsARF5*.

The phenotypes of *OE1* and *OE2* plants were compared to that of wild type (*Col-0*), heterozygous/homozygous *Arabidopsis* mutants of *AtARF5* (*arf-/+* and *arf-/-*, respectively). Our results (summarized in **Table 3**) showed that the *OE1* plants were significantly taller (35.17 ± 3.24 cm in height) with broader leaves (9.76 ± 1.08 mm) compared to the *Col-0* plants. Such an enhancement in the growth of the *OE1* plants was accompanied by well-developed vascular tissues. Thus, these results showed that overexpressing *MsARF5* promotes plant growth. We also overexpressed *MsARF5* in the homozygous *arf-/-* mutant (designated as *OE2*) and found that the transgenic plants resembled the heterozygous mutant phyenotypically. In addition, *OE2* outperformed the *arf-/-* mutant in multiple plant growth parameters such as plant height, rosette leaf diameter, and leaf width. This suggested that *MsARF5* was able to rescue the *Arabidopsis arf 5* mutant in vegetative growth.

**Table 3:** Phenotypic traits of transgenic *A. thaliana*.

Plant type	Height/cm	Basal diameter/mm	Rosette diameter/mm	Leaf width/mm	Internode length/cm
*Col-0*	28.47 ± 2.01b	0.67 ± 0.07abc	60.05 ± 3.09a	6.44 ± 0.37b	2.53 ± 0.23a
*arf-/+*	22.84 ± 1.62c	0.64 ± 0.09bc	42.23 ± 2.31c	6.34 ± 0.45b	2.20 ± 0.33ab
*arf-/-*	14.27 ± 1.60d	0.58 ± 0.08cd	48.47 ± 3.22bc	5.88 ± 0.61b	2.07 ± 0.19b
*OE1*	35.17 ± 3.24a	0.78 ± 0.08ab	56.44 ± 6.32ab	9.76 ± 1.08a	2.71 ± 0.26a
*OE2*	19.21 ± 2.14c	0.48 ± 0.06d	54.63 ± 3.08b	6.57 ± 0.39b	2.04 ± 0.17b

The letters in the table are the results of Duncan’s multiple comparisons. The same letter in each column indicates that the difference is not significant, and different letters indicate that the difference is significant P = 0.05.

### 3.5 Impact of *MsARF5* overexpression on flowering and seed development

We next investigated the impact of *MsARF5* overexpression on flowering and seed development. Compared to *Col-0*, the homozygous mutant *arf-/-* plants showed a delay in leaf development and bolting (**Table 4**). No flowering was observed in the homozygous mutant *arf-/-* plants. By contrast, the *OE1* and the *OE2* plants show no difference compared to the *Col-0* except that *OE1* had a longer life cycle. Seed phenotypes in various *Arabidopsis* lines were summarized in **Table 5**. In line with the notion that insufficient *AtARF5* expression could lead to defects in seed development, we found that ~12.5% of the *arf-/+* plants showed abnormal development (**Figure 4D**). Although overexpressing *MsARF5* in *Arabidopsis* allowed the flower formation in the *arf-/-* mutant, significant differences in seedpod length, seed number, and the aspect ratio were found between the *arf-/*+ and *Col-0* plants. Except for *OE2*, the seed number of *OE1*, WT and *arf-/+* was inversely proportional to the growth cycle. It can be speculated that the overexpression of *MsARF5* can preferentially promote the vegetative growth of plants. However, *arf-/-* seeds could not develop normally.

**Table 4:** Phenotypes of transgenic *A. thaliana*.

Plant type	Leaf development/d	Bolting/d	Flowering/d	Stop high growth/d
*Col-0*	5.70 ± 0.70c	37.97 ± 0.81c	40.3 ± 0.47c	67.67 ± 3.36c
*arf-/+*	6.30 ± 0.53c	47.77 ± 1.10a	51.73 ± 0.91a	82.13 ± 3.15a
*arf-/-*	8.07 ± 0.64ab	49.53 ± 1.27a	–	65.10 ± 3.26c
*OE1*	6.60 ± 1.19bc	38.63 ± 0.89c	40.77 ± 0.68bc	76.06 ± 2.42b
*OE2*	5.60+0.50c	38.36 ± 1.45c	41.80 ± 0.81b	69.30 ± 2.14c

The letters in the table are the results of Duncan’s multiple comparisons. The same letter in each column indicates that the difference is not significant, and different letters indicate that the difference is significant P = 0.05.

**Table 5:** Seed characteristics for different lines of *A. thaliana*.

Plant type	Seed pods length/mm	Number	Aspect ratio	Germination rate %	Time/d
*Col-0*	0.74 ± 0.04a	28.4 ± 1.67a	1.84 ± 0.17b	98.88 ± 0.83a	4.4 ± 0.48b
*arf-/+*	0.62 ± 0.09ab	18.0 ± 1.41b	2.08 ± 0.23ab	97.13 ± 1.13a	4.1 ± 0.36b
*arf-/-*	–	–	–	–	–
*OE1*	0.72 ± 0.06ab	21.2 ± 2.68b	1.65 ± 0.15b	93.87 ± 1.96b	4.2 ± 0.42b
*OE2*	0.63 ± 0.06b	10.4 ± 2.61c	2.30 ± 0.22a	65.71 ± 5.59c	6.0 ± 0.67a

The letters in the table are the results of Duncan’s multiple comparisons. The same letter in each column indicates that the difference is not significant, and different letters indicate that the difference is significant P = 0.05.

### 3.6 Response to exogenous auxin in transgenic plants

**Figure 5:**
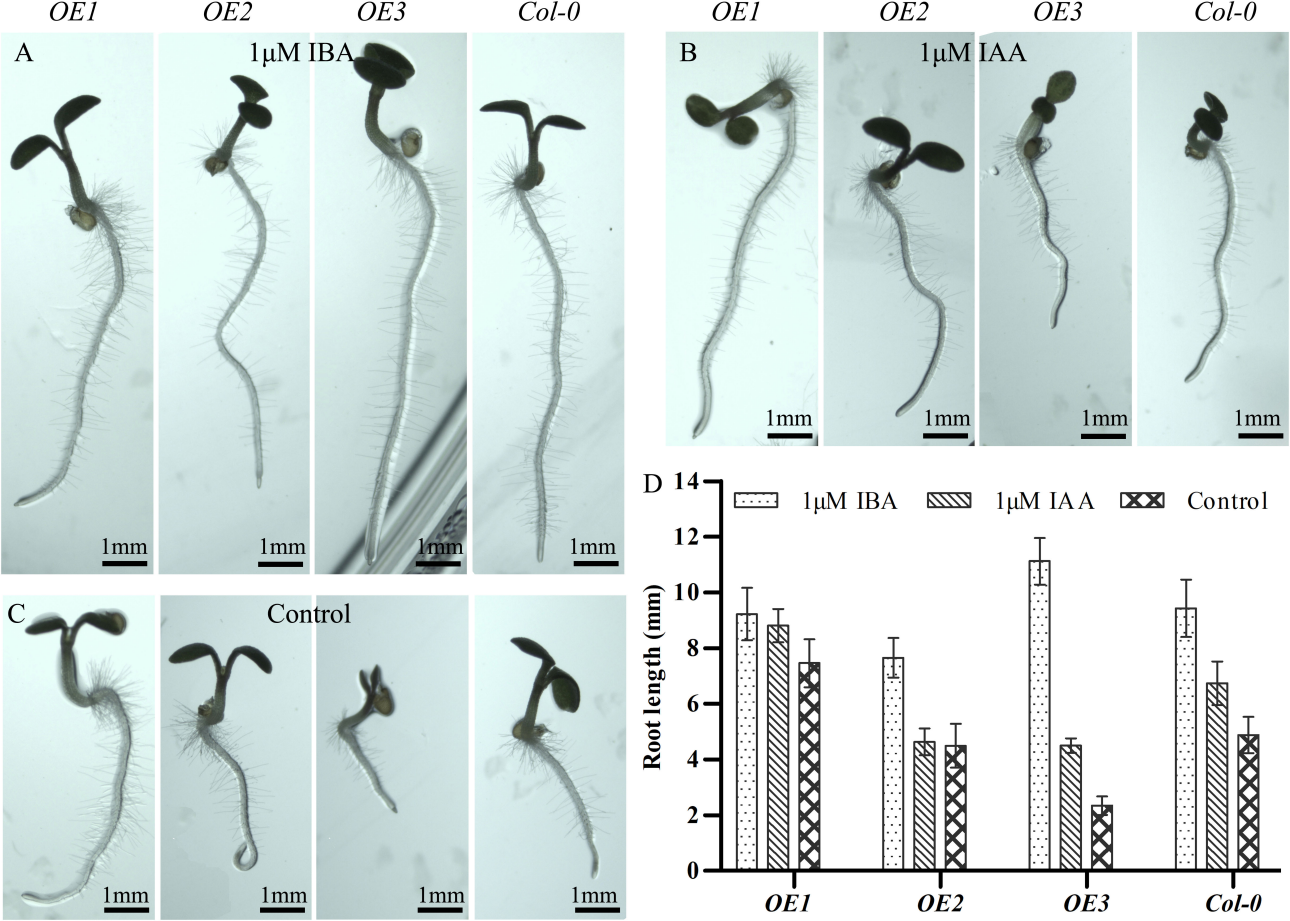
Response to exogenous auxin in transgenic *Arabidopsis* overexpressing *MsARF5*.**(A–C)**. Impact of exogenous IBA **(A)** and IAA **(B)** on transgenic seedlings overexpressing *MsARFs* compared to controls **(C)**. **(D)** Quantitative analysis on root length following exposure to IBA/IAA. The letters in the figure are the results of Duncan’s multiple comparisons. The same letter in each column indicates that the difference is not significant, and different letters indicate that the difference is significant P = 0.05.

Next, we determined the response to IAA and indole-3-butyric acid (IBA) in *Arabidopsis* plants of different genetic backgrounds (**Figure 5**). For this experiment, we also created a transgenic line in which the expression of *MsARF5* is driven by the native promoter (designated as *OE3*). First, we observed that both IAA and IBA promoted the primary root elongation in the *Col-0*, *OE1*, and *OE3* plants (**Figures 5A-C**). Notably, the impact of IBA was far more significant than that of IAA, especially in the *OE3* plants. Moreover, a significant increase in root elongation was observed in the *OE2* plants following exposure to IBA, but not IAA (**Figure 5D**). This indicates that *MsARF5* is sensitive to exogenous auxin, and the response to 1 μM IBA is higher than that of 1 μM IAA. These results also verified that *ARF5* is an essential gene for seed embryo morphogenesis under auxin signaling.

## 4 Discussion

Here we found 4 groups of ARFs in *M. sieboldii*, Class I–IV. Each class has a counterpart in *M Sieboldii* with Class I–III in *A. thaliana* corresponding to Class V, I and II, respectively. Similar analysis also identified pairing class of ARFs between *M. sieboldii* and *M. truncatula*. Interestingly, *M. truncatula* showed 8 groups of *ARFs* and class VIII may be a result of hybridization during evolution (Shen et al., 2015). We noted that no MsARF protein clustered with AtARF12-15,21-23. These genes form a gene cluster on Arabidopsis chromosome I and are mainly expressed during embryogenesis and seed development (Okushima et al., 2005). While there is no similar gene form AtARF12-15,21-23 in *M. Sieboldii and O. sativa*. In addition, MsARFs proteins often cluster independently, or at the periphery of monocotyledons and dicotyledons, indicating that in terms of evolutionary relationship, *M. Sieboldii* may be more primitive. The update of APG IV shows that Magnoliaceae belongs to the base of angiosperms, and its evolutionary relationship is earlier than that of monocotyledons and dicotyledons (Byng et al., 2016). This also supports the results of phylogenetic analysis in this paper.

We identified 25 *ARF* genes in *M. sieboldii via* homology searching. The encoded proteins could act as either transcriptional activators or repressors, depending on the amino acid compositions in the MR region. Previous studies using transfected protoplasts showed transcriptional activation activity of Q-rich ARFs in *Arabidopsis* (Tiwari et al., 2003). Later, ARFs proteins with different transcriptional activities had also been identified in non-model plants. For instance, ARFs with MRs that are rich in serine (S), proline (P), and glutamine (Q) exhibit transcriptional repressing activities in *Dimocarpus longan* (Lin et al., 2013). Similarly, ARFs with MRs rich in Q/S/L(leucine) function as activators in multiple species including *Prumus persica* (Shi et al., 2012)、*Vitis vinifera* (Yuan et al., 2015), *Lycopersicon esculentum* (Wu, 2011), and *Malus pumila* (Li et al., 2015). In our study, *Ms*ARF5 was predicted as an activator due to the same amino acid composition as found in transcriptional activators in *M. pumila*. In addition, we also found that all *Ms*ARFs with Q-rich MRs belong to Class II.

The involvement of auxin in vascular differentiation had been demonstrated decades ago (Aloni, 1995; Mattsson et al., 2003). Active transportation of auxin and an elevated concentration had been found in regions where vascular differentiation occurs. In addition, impaired patterning in vascular differentiation had been found in various mutants that are defective in the biosynthesis, transportation, or signaling of auxin. At the molecular level, *MP*/*AtARF5* had been identified as a central regulator in leaf vascularization (Krogan and Berleth, 2012; Krogan et al., 2012) and root growth (Krogan et al., 2016). In *M. sieboldii*, vascular tissue differentiation was preliminarily observed after stratification for 30 days. Notably, the expression of *MsARF5* also showed a significant increase at this time, indicating a role of *MsARF5* in regulating vascular tissue differentiation in the seeds.

In addition to vascular differentiation, *ARF5/MP* also plays an essential role in embryo development as the *Arabidopsis arf/mp* mutant is impaired in flower organ formation and seed development (Przemeck et al., 1996; Hardtke and Berleth, 1998). In this study, we not only confirmed the infertility of the *arf/mp* mutant but also showed that the *MsARF5*-overexpressing line is also infertile. While no flower organ formation was observed in the former, the latter exhibited normal development of floral organs and pod but no seeds. That fact either complete loss-of-function or overexpression leads to abnormal embryo development suggests that the expression level of *ARF5* controls the final phenotype. These observations also indicated a similar function of *MsARF5* and its homolog in *Arabidopsis* (Schlereth et al., 2010; Krogan et al., 2016).

Our finding on the two different phenotypes in the *MsARF5*-overexpressing *Arabidopsis* plants indicated the expression level of *ARF5* is behind the final plant phenotype. Specifically, *OE1* and *OE2* showed significant altered vegetative growth including height, basal diameter, and leaf width compared to WT plants. These observations are in line with previous findings that *ARF5/MP* in *Arabidopsis* is a quantitative gene and that it plays an essential role in mediating auxin signaling and its polar transport (Krogan et al., 2016). The patterning events in embryos were under the direct control of *ARF5/MP-*mediated auxin signaling and transportation. Accordingly, *ARF5/MP* acts as a switch to regulate the expression of *PIN* (pin formed) auxin efflux facilitators during pattern formation of root and shoot (Wenzel et al., 2007). This is further supported by previous studies showing the binding of *ARF5/MP* on the promoters of multiple *PIN* genes (Okushima et al., 2005). Together, our data indicate a role of *MsARF5* in mediating auxin signaling and transportation to control the patterning of multiple plant organs.

## 5 Conclusions

A total of 25 ARF genes were identified in *M sieboldii*. Diversity among these genes was found due to the presence of different functional domains, suggesting diversity in function. Gene expression analysis of *MsARF5* showed a similar pattern to that of auxin during seed germination. The function of *MsARF5* was further investigated by overexpressing in *Arabidopsis*, which showed an increase in the growth of vascular tissues and promotion of seed germination. In addition, *MsARF5* can rescue the deficiency in flowering of the *arf* mutant.

## Data availability statement

The data presented in the study are deposited in the National Genomics Data Center (NGDC) repository, accession number PRJCA009763.

## Ethics statement

*M. sieboldii* seeds were used in this study. *M. sieboldii* is a National level 3 protection plants of China and the identification information is stored in the Plant Photo Bank of China (PPBC ID: 2657292). The seed sample was kindly provided by Benxi Botanical Garden (Benxi, Liaoning, China). We complied with all the relevant institutional, national and international guidelines.

## Author contributions

XL conceived the experiment. MM and LL collected resources. MM conducted the experiment. MM and WA analyzed the results. MM wrote the manuscript. XL and XX revised the manuscript. All authors contributed to the article and approved the submitted version.

## Funding

This research and the APC was funded by National Natural Science Foundation of China, grant number 31570621 and 32101508.

## Acknowledgments

We thank Prof. Yongxiu Liu (Institute of Botany, Chinese Academy of Sciences) for providing the T-DNA mutant of *ARF5* (salk_001058) seeds. All sequencing was outsourced to Novogene Bioinformatics Institute, Beijing, China. We thank Novogene Co., Ltd. for the technology support.

## Conflict of interest

The authors declare that the research was conducted in the absence of any commercial or financial relationships that could be construed as a potential conflict of interest.

## Publisher’s note

All claims expressed in this article are solely those of the authors and do not necessarily represent those of their affiliated organizations, or those of the publisher, the editors and the reviewers. Any product that may be evaluated in this article, or claim that may be made by its manufacturer, is not guaranteed or endorsed by the publisher.

## Supplementary material

Supplementary Figure 1 Chromosomal distribution of *MsARFs*.

Supplementary Figure 2 Gene structure analysis and motif characterization of *MsARFs*.

Supplementary Figure 3 Identification of ARFs in *M. sieboldii*, *Medicago truncatula*, *Vitis vinifera* and apple. The unrooted tree was constructed using MEGA-X with the neighbor-joining method (bootstrap of 1000).

Supplementary Table 1 PCR primer names and sequences.

## References

[B1] Ai W. Liu Y. Mei M. Zhang X. Tan E. Liu H.. (2022). A chromosome-scale genome assembly of the Mongolian oak (*Quercus mongolica*). Mol. Ecol. Resour. 22 2396–2410. doi: 10.1111/1755-0998.13616 35377556

[B2] Aloni R. (1995). The induction of vascular tissues by auxin and cytokinin Plant Hormones (Kluwer Academic) 531–546. doi: 10.1007/978-94-011-0473-9_25

[B3] Belin C. Megies C. Hauserova E. Lopez-Molina L. (2009). Abscisic acid represses growth of the arabidopsis embryonic axis after germination by enhancing auxin signaling. Plant Cell 21, 2253–2268. doi: 10.1105/tpc.109.067702 19666738 PMC2751952

[B4] Boter M. Calleja-Cabrera J. Carrera-Castao G. Wagner G. Oate-Sánchez L. (2019). An integrative approach to analyze seed germination in brassica napus. Front. Plant Sci. 10, 1342. doi: 10.3389/fpls.2019.01342 31708951 PMC6824160

[B5] Byng J. W. Chase M. W. Christenhusz M. J. M. Fay M. F. Judd W. S. (2016). An update of the angiosperm phylogeny group classification for the orders and families of flowering plants: APG IV. Botanical J. Linn. Soc. 181, 1–20. doi: 10.1111/boj.12385

[B6] Chen C. Chen H. Zhang Y. Thomas H. R. Frank M. H. He Y.. (2020). TBtools: An integrative toolkit developed for interactive analyses of big biological data. Mol. Plant 13, 1194–1202. doi: 10.1016/j.molp.2020.06.009 32585190

[B7] Clough S. J. Bent A. F. (1998). Floral dip: a simplified method for agrobacterium-mediated transformation of arabidopsis thaliana. Plant J. 16, 735–743. doi: 10.1046/j.1365-313x.1998.00343.x 10069079

[B8] Cole M. Chandler J. Weijers D. Jacobs B. Comelli P. Werr W. (2009). DORNROSCHEN is a direct target of the auxin response factor MONOPTEROS in the arabidopsis embryo. Development 136, 1643–1651. doi: 10.1242/dev.032177 19369397

[B9] Donner T. J. Sherr I. Scarpella E. (2009). Regulation of preprocambial cell state acquisition by auxin signaling in arabidopsis leaves. Development 136, 3235–3246. doi: 10.1242/dev.037028 19710171

[B10] Finn R. D. Clements J. Eddy S. R. (2011). HMMER web server: interactive sequence similarity searching. Nucleic Acids Res. 39, 29–37. doi: 10.1093/nar/gkr367 PMC3125773 21593126

[B11] Galstyan A. Nemhauser J. L. (2019). Auxin promotion of seedling growth *via* ARF5 is dependent on the brassinosteroid-regulated transcription factors BES1 and BEH4. Plant Direct 3, e00166. doi: 10.1002/pld3.166 31508562 PMC6722427

[B12] Hardtke C. S. Berleth T. (1998). The arabidopsis gene MONOPTEROS encodes a transcription factor mediating embryo axis formation and vascular development. EMBO J. 17, 1405–1411. doi: 10.1093/emboj/17.5.1405 9482737 PMC1170488

[B13] Krogan N. T. Berleth T. (2012). A dominant mutation reveals asymmetry in MP/ARF5 function along the adaxial-abaxial axis of shoot lateral organs. Plant Signaling Behav. 7, 940–943. doi: 10.4161/psb.20790 PMC3474690 22751359

[B14] Krogan N. T. Ckurshumova W. Marcos D. Caragea A. E. Berleth T. (2012). Deletion of MP/ARF5 domains III and IV reveals a requirement for Aux/IAA regulation in arabidopsis leaf vascular patterning. New Phytol. 194:391–401. doi: 10.1111/j.1469-8137.2012.04064.x 22320407

[B15] Krogan N. T. Marcos D. Weiner A. I. Berleth T. (2016). The auxin response factor MONOPTEROS controls meristem function and organogenesis in both the shoot and root through the direct regulation of PIN genes. New Phytol. 212, 45–50. doi: 10.1111/nph.14107 PMC5596637 27441727

[B16] Kumar S. Stecher G. Li M. Knyaz C. Tamura K. (2018). MEGA X: Molecular evolutionary genetics analysis across computing platforms. Mol. Biol. Evol. 35, 1547–1549. doi: 10.1093/molbev/msy096 29722887 PMC5967553

[B17] Larkin M. A. Blackshields G. Brown N. P. Chenna R. Mcgettigan P. A. Mcwilliam H.. (2007). Clustal W and clustal X version 2.0. Bioinformatics 23, 2947–2948. doi: 10.1093/bioinformatics/btm404 17846036

[B18] Lei Y. (2007). Cloning and expression of the resveratrol synothase gene from polygonum cuspidatum (Beijing:Chinese Academy of Agricultural Sciences).

[B19] Li P. Lu X. Yao F. Guo R. (2006). Preliminary study on reasons of seed dormancy of magnolia sieboldii K.Koch. Seed 25:36–39. doi: 10.16590/j.cnki.1001-4705.2006.02.045

[B20] Lin L. X. Lin Y. L. Lai Z. X. (2013). Analyses of DIARF5-1 expression levels by qPCR under the treatment of different hormones in longan. Horticult. Seed 10 17–20. doi: 10.3969/j.issn.2095-0896.2013.10.005

[B21] Li H. F. Ran K. He P. Wang H. B. Chang Y. S. Sun Q. R.. (2015). Genome-wide identification and expression analysis of auxin response factor (ARF) gene family in apple. Plant Physiol. J 51:1045–1054. doi: 10.13592/j.cnki.ppj.2015.0173

[B22] Liscum E. Reed J. W. (2002). Genetics of Aux/IAA and ARF action in plant growth and development. Plant Mol. Biol. 49, 387–400. doi: 10.1023/A:1015255030047 12036262

[B23] Liu X. Zhang H. Zhao Y. Feng Z. Li Q. Yang H. Q.. (2013). Auxin controls seed dormancy through stimulation of abscisic acid signaling by inducing ARF-mediated ABI3 activation in arabidopsis. Proc. Natl. Acad. Sci. United States America 110, 15485–15490. doi: 10.1073/pnas.1304651110 PMC3780901 23986496

[B24] Lu X. Mei M. Liu Y. Zhang X. Ma B. (2014). Effect of treatment with GA_3 and variable temperature stratification on germination and endogenous hormones of magnolia sieboldii seeds. Acta Botanica Boreali-Occidentalia Sin 34:1828–1835. doi: 10.7606/j.issn.1000-4025.2014.09.1828

[B25] Mattsson J. Ckurshumova W. Berleth T. (2003). Auxin signaling in arabidopsis leaf vascular development. Plant Physiol. 131, 1327–1339. doi: 10.1104/pp.013623 12644682 PMC166892

[B26] Mei M. Lu X. J. Zhang X. L. Liu G. L. Sun X. M. (2017). Variation in carbohydrates and screening of related differential proteins during the seed germination of magnolia sieboldii k. Koch. Trees 31, 63–75. doi: 10.1007/s00468-016-1456-8

[B27] Mei M. Wei J. Ai W. Zhang L. Lu X. J. (2021). Integrated RNA and miRNA sequencing analysis reveals a complex regulatory network of magnolia sieboldii seed germination. Sci. Rep. 11, 10842. doi: 10.1038/s41598-021-90270-y 34035372 PMC8149418

[B28] Moller B. Weijers D. (2009). Auxin control of embryo patterning. Cold Spring Harbor Perspect. Biol. 1, a001545. doi: 10.1101/cshperspect.a001545 PMC2773644 20066117

[B29] Odat O. Gardiner J. Sawchuk M. G. Verna C. Donner T. J. Scarpella E. (2014). Characterization of an allelic series in the MONOPTEROS gene of arabidopsis. Genesis 52, 127–133. doi: 10.1002/dvg.22729 24281793

[B30] Okushima Y. Overvoorde P. J. Arima K. Alonso J. M. Chan A. Chang C.. (2005). Functional genomic analysis of the AUXIN RESPONSE FACTOR gene family members in arabidopsis thaliana: Unique and overlapping functions of ARF7 and ARF19. Plant Cell 17, 444–463. doi: 10.1105/tpc.104.028316 15659631 PMC548818

[B31] Przemeck G. K. Mattsson J. Hardtke C. S. Sung Z. R. Berleth T. (1996). Studies on the role of the arabidopsis gene MONOPTEROS in vascular development and plant cell axialization. Planta 200, 229–237. doi: 10.1007/BF00208313 8904808

[B32] Quint M. Gray W. M. (2006). Auxin signaling. Curr. Opin. Plant Biol. 9, 448–453. doi: 10.1016/j.pbi.2006.07.006 16877027 PMC2424235

[B33] Rademacher E. H. Lokerse A. S. Schlereth A. Llavata-Peris C. I. Bayer M. Kientz M.. (2012). Different auxin response machineries control distinct cell fates in the early plant embryo. Dev. Cell 22, 211–222. doi: 10.1016/j.devcel.2011.10.026 22264733

[B34] Rousselin P. Kraepiel Y. Maldiney R. Miginiac and E. (1992). Characterization of three hormone mutants of nicotiana plumbaginifolia: evidence for a common ABA deficiency. Theor. Appl. Genet. 85, 213–221. doi: 10.1007/BF00222862 24197307

[B35] Schlereth A. Möller B. Liu W. Kientz M. Flipse J. Rademacher E. H.. (2010). MONOPTEROS controls embryonic root initiation by regulating a mobile transcription factor. Nature 464, 913–916. doi: 10.1038/nature08836 20220754

[B36] Shen C. Yue R. Sun T. Zhang L. Xu L. Tie S.. (2015). Genome-wide identification and expression analysis of auxin response factor gene family in medicago truncatula. Front. Plant Sci. 6, 73. doi: 10.3389/fpls.2015.00073 25759704 PMC4338661

[B37] Shi M. Li Y. Zhang W. Liu Y. (2012). Progress in mechanism of auxin response factors. Biotechnol. Bull. 8 24–28. doi: 10.13560/j.cnki.biotech.bull.1985.2012.08.028

[B38] Tiwari S. B. Hagen G. Guilfoyle T. (2003). The roles of auxin response factor domains in auxin-responsive transcription. Plant Cell 15, 533–543. doi: 10.1105/tpc.008417 12566590 PMC141219

[B39] Ulmasov T. Hagen G. Guilfoyle T. J. (1997). ARF1, a transcription factor that binds to auxin response elements. Science 276, 1865–1868. doi: 10.1126/science.276.5320.1865 9188533

[B40] Vidaurre D. P. Ploense S. Krogan N. T. Berleth T. (2007). AMP1 and MP antagonistically regulate embryo and meristem development in arabidopsis. Development 134, 2561–2567. doi: 10.1242/dev.006759 17553903

[B41] Wang C. K. Han P. L. Zhao Y. W. Yu J. Q. You C. X. Hu D. G.. (2020). Genome-wide analysis of auxin response factor (ARF) genes and functional identification of MdARF2 reveals the involvement in the regulation of anthocyanin accumulation in apple New Zealand Journal of Crop and Horticultural Science 49, 78–91. doi: 10.1080/01140671.2020.1779756

[B42] Wang L. Hua D. He J. Duan Y. Chen Z. Hong X.. (2011). Auxin response Factor2 (ARF2) and its regulated homeodomain gene HB33 mediate abscisic acid response in arabidopsis. PloS Genet. 7, e1002172. doi: 10.1371/journal.pgen.1002172 21779177 PMC3136439

[B43] Wang D. Pei K. Fu Y. Sun Z. Li S. Liu H.. (2007). Genome-wide analysis of the auxin response factors (ARF) gene family in rice (Oryza sativa). Gene 394, 13–24. doi: 10.1016/j.gene.2007.01.006 17408882

[B44] Wan S. Li W. Zhu Y. Liu Z. Zhan J. (2014). Genome-wide identification, characterization and expression analysis of the auxin response factor gene family in vitis vinifera. Plant Cell Rep. 33, 1365–1375. doi: 10.1007/s00299-014-1622-7 24792421

[B45] Wenzel C. L. Schuetz M. Yu Q. Mattsson J. (2007). Dynamics of MONOPTEROS and PIN-FORMED1 expression during leaf vein pattern formation in arabidopsis thaliana. Plant J. 49, 387–398. doi: 10.1111/j.1365-313X.2006.02977.x 17217464

[B46] Woodward A. W. Bartel B. (2005). A receptor for auxin. Plant Cell 17, 2425–2429. doi: 10.1105/tpc.105.036236 16141189 PMC1197424

[B47] Wu J. (2011). Identification and expression profile analysis of auxin response genes and preliminary functional characterization of SlARF5 in tomato (Solanum lycopersicum) (Zhejiang University, Hangzhou: College of Agriculture and Biotechnology).

[B48] Xing H. Pudake R. N. Guo G. Xing G. Hu Z. Zhang Y.. (2011). Genome-wide identification and expression profiling of auxin response factor (ARF) gene family in maize. BMC Genomics 12, 178. doi: 10.1186/1471-2164-12-178 21473768 PMC3082248

[B49] Yuan H. Zhao M. Wu W. Yu H. Qian Y. Wang Z.. (2015). Genome-wide identification and expression analysis of auxin-related gene families in grape. Yi Chuan 37, 720–730. doi: 10.16288/j.yczz.14-390 26351172

[B50] Yu Y. Z. Du J. Wang G. Ji J. (2003). Studies on the freeze-thaw method of transforming recombinant plasmid DNA into agrobacterium tumefaciens. J. Jilin Agric. Univ. 25, 257–259, 262. doi: 10.3969/j.issn.1000-5684.2003.03.006

[B51] Zhao Z. Andersen S. U. Ljung K. Dolezal K. Miotk A. Schultheiss S. J.. (2010). Hormonal control of the shoot stem-cell niche. Nature 465, 1089–1092. doi: 10.1038/nature09126 20577215

