# Supplementary material for: Genome-wide identification of the auxin response factor (ARF) gene family in Magnolia sieboldii and functional analysis of MsARF5
Source: Front Plant Sci. 2022 Oct 5;13:958816. doi: 10.3389/fpls.2022.958816 (PMC9581218; doi:10.3389/fpls.2022.958816)
Supplement: Supplementary Figure 1 — Chromosomal distribution of MsARFs. [file DataSheet_1.pdf]

Supplementary Material:

Genome-wide Identification of the Auxin Response Factor (ARF) Gene Family in *Magnolia sieboldii* and Functional Analysis of *MsARF5*

Mei Mei[1,2], Wanfeng Ai[1], Lin liu[1], Xin Xu[1], Xiujun Lu[1*]

[1]Department of Forestry, Shenyang Agricultural University, Shenyang, China

[2]Biotechnology and Analysis Test Center, Liaoning Academy of Forest Science, Shenyang, China

*** Correspondence:**
Xiujun Lu
lxjsyau@syau.edu.cn

List of Figures

Supplemental Figure 1. Chromosomal distribution of MsARFs. 2

Supplemental Figure 2. Gene structure analysis and motif characterization of *MsARFs*. 3

Supplemental Figure 3. Identification of ARFs in *M. sieboldii*, *Medicago truncatula*, *Vitis vinifera* and apple. The unrooted tree was constructed using MEGA-X with the neighbor-joining method (bootstrap of 1000). 4

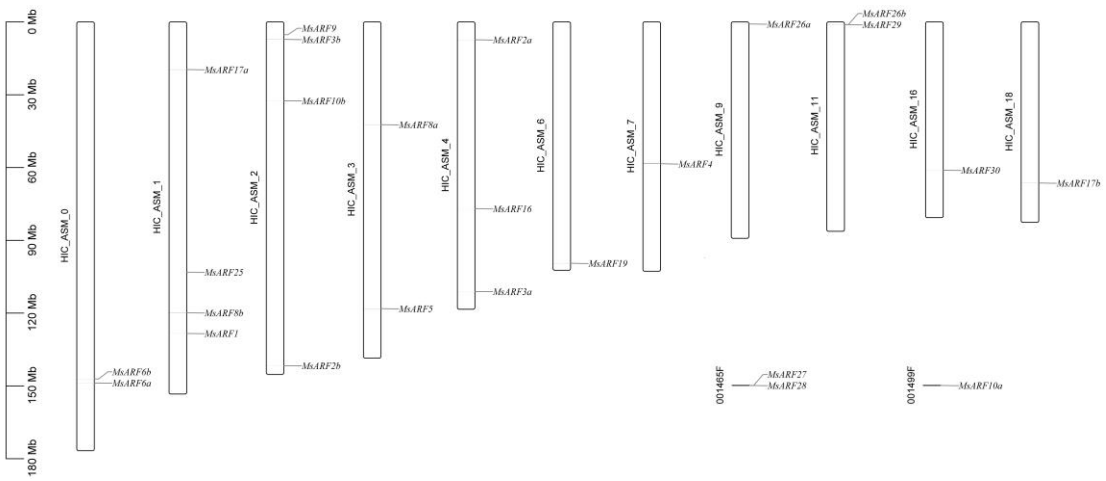

Supplemental Figure 1. Chromosomal distribution of *MsARFs*.

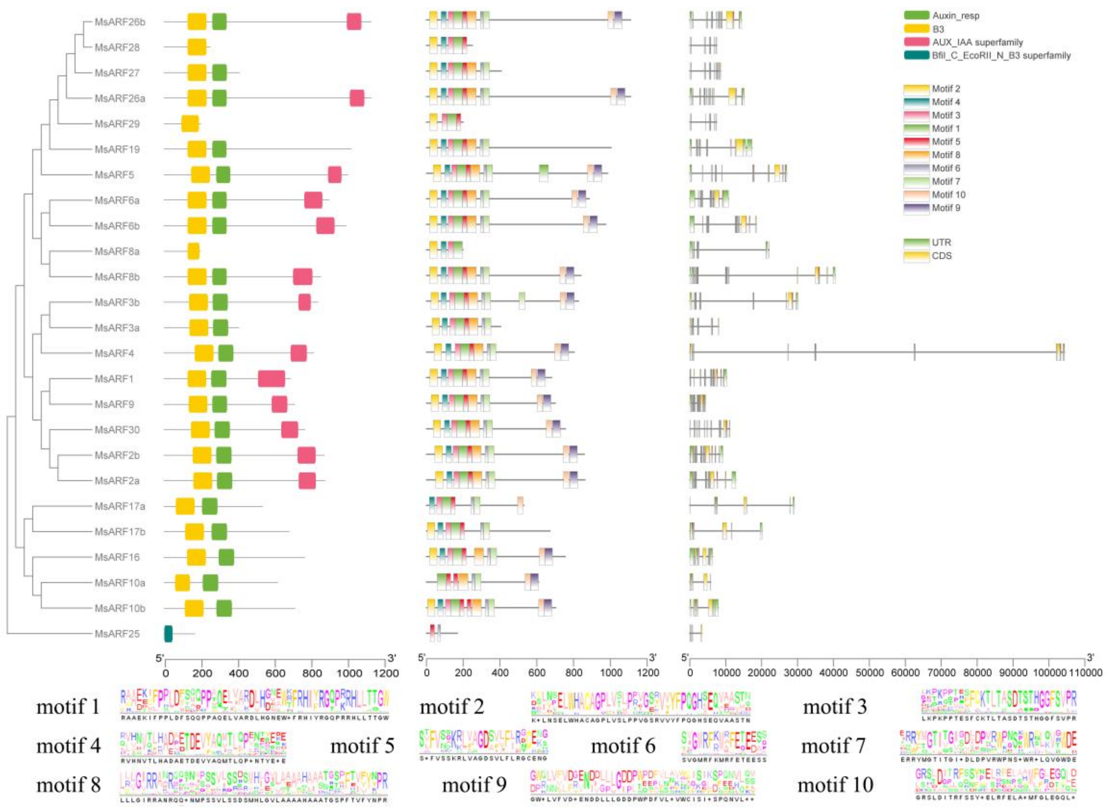

Supplemental Figure 2. Gene structure analysis and motif characterization of *MsARFs*.

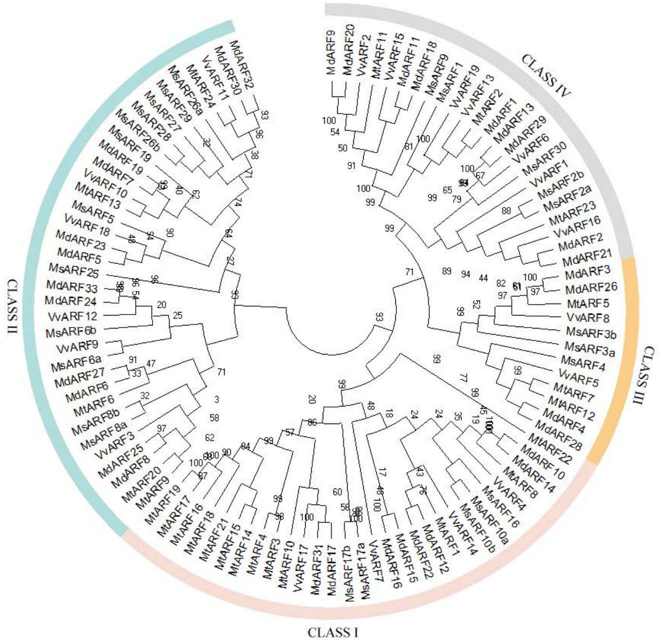

Supplemental Figure 3. Identification of ARFs in *M. sieboldii*, *Medicago truncatula*, *Vitis vinifera* and apple. The unrooted tree was constructed using MEGA-X with the neighbor-joining method (bootstrap of 1000).
