# Supplementary material for: Genome-wide identification of the auxin response factor (ARF) gene family in Magnolia sieboldii and functional analysis of MsARF5
Source: Front Plant Sci. 2022 Oct 5;13:958816. doi: 10.3389/fpls.2022.958816 (PMC9581218; doi:10.3389/fpls.2022.958816)
Supplement: Supplementary Table 1 — PCR primer names and sequences. [file Table_1.docx]

**Supplemental Table 1. PCR primer names and sequences**

| **Name** | **Sequence** |
| --- | --- |
| qPCR-MsARF5-F | TTGCCATCATCCGTTCTCTC |
| qPCR-MsARF5-R | TCTGCGTTTACCTGACTCCTCT |
| AtACT7-F | GGTGAGGATATTCAGCCACTTGTCTG |
| AtACT7-R | ACCATGACACCAGTGTGCCT |
| MsACT7-F | TCAACCCCAAAGCAAACAGA |
| MsACT7-R | CCGCAAGATCCAAACGAA |
| MsARF5-F | ATGTCTTCTGTTCAAGAGAACTGC |
| MsARF5-R | TCATGGGCCATCCCAAACATC |
| proA-F | GACCTGCAGGCATGCAAGCTTTCAGTGTGGGCTATGGAAGGC |
| pro-R | TTACCCTCAGATCTACCATGGGCAGTTCTCTTGAACAGAAGACATG |
